# Insights into the Pathophysiology of Scheuermann’s Kyphosis: From Structural Deformities to Genetic Predisposition and Underlying Signalling Pathways

**DOI:** 10.3390/biom16010056

**Published:** 2025-12-30

**Authors:** Angelos Kaspiris, Ioannis Spyrou, Vasileios Marougklianis, Spyridoula Roberta Afrati, Evangelos Sakellariou, Iordanis Varsamos, Panagiotis Karampinas, Elias Vasiliadis, Spiros G. Pneumaticos

**Affiliations:** Third Department of Orthopaedic Surgery, School of Medicine, National and Kapodistrian University of Athens, “KAT” General Hospital, Nikis 2, 14561 Athens, Greece; ispyrou@med.uoa.gr (I.S.); jordan.var1995@gmail.com (I.V.);

**Keywords:** Scheuermann’s kyphosis, mechanical factors, vertebral body wedging, bone metabolism, endochondral ossification, intervertebral disc, genetic link, defective mechanosensory signalling

## Abstract

Scheuermann’s kyphosis (SK) is a rigid dorsal kyphosis of unclear pathophysiological origin. The aim of this review is to summarise current theories and both clinical and experimental findings regarding the underlying mechanisms of SK. Emerging evidence highlights the significant role of excessive mechanical loading as a major contributor to defective growth of the cartilaginous vertebral endplate. This is associated with the formation of Schmorl’s nodes, disruption of the ring apophysis, and compromised intervertebral disc integrity—ultimately resulting in vertebral body wedging and thickening of the anterior longitudinal ligament. In addition, numerous studies have investigated the genetic contribution and underlying molecular mechanisms involved in the pathogenesis of SK. Recent in vivo findings suggest an association between asymmetric mechanosensory activation of cerebrospinal fluid (CSF), contacting neurons, and defective Reissner fibre signalling, which may contribute to abnormal spinal morphogenesis in the sagittal thoracic plane. These findings indicate a potential link between altered CSF dynamics and the development of SK. Taken together, the evidence supports a multifactorial aetiology, with both genetic and biomechanical factors playing central roles in the development of Scheuermann’s kyphosis. The interpretation of the underlying pathophysiological mechanism could result in the early detection of the subjects that may have genetical predisposition for SK appearance and the development of target molecular treatments in order to counter the progression of the deformity.

## 1. Introduction

Scheuermann’s Kyphosis (SK) is the most common form of structural kyphosis in adolescence [1], typically manifesting between 12 and 15 years of age [2]. It presents as a rigid kyphotic deformity, often accompanied by pain and cosmetic complaints, and is diagnosed based on the radiographic criteria established by Sørensen in 1964 [3]. These require at least three consecutive vertebrae wedged by five degrees or more at the apex of the deformity, resulting in thoracic hyperkyphosis [3]. Several modified diagnostic criteria have been proposed, including thoracic kyphosis > 45° with at least one wedged vertebra, kyphosis with at least two wedged vertebrae, or kyphosis ≥ 35° with one wedged vertebra. Some authors also consider the presence of characteristic radiographic abnormalities in a single wedged vertebra regardless of kyphotic angle. However, these criteria are inconsistently applied across studies, making comparisons difficult [4,5,6,7]. Scheuermann’s Kyphosis is commonly associated with irregular vertebral endplates, Schmorl’s nodes, and disc impairment or loss of disc space height [8]. The Scoliosis Research Society defines the upper limit of normal thoracic kyphosis at 45 degrees. The inconsistent diagnostic criteria, along with the variability in defining the upper limit of normal thoracic kyphosis, poses a diagnostic challenge and underscores the importance of the characteristic radiographic appearance of the disease. Atypical SK is rarer, with the vertebral changes occurring lower in the thoracolumbar or lumbar region of the spine. It was originally reported in young athletic patients and a classification was proposed by Blumenthal et al. [9]. Regarding epidemiology, the reported prevalence of SK varies widely across studies, ranging from 0.4% to 10% [10]. A large multinational study of over 10,000 patients reported a prevalence of 8% with equal distribution between sexes, although prevalence varied by country [6]. Differences in epidemiological reporting may also be partially attributed to the variability of diagnostic criteria.

Although the exact aetiopathogenesis of SK remains undetermined, both mechanical and genetic factors appear to play crucial roles in the development of the deformity. The degree of involvement as well as the interaction between the proposed causative factors is also elusive. Over the years, several theories have been proposed, each focusing primarily on a specific pathophysiological mechanism to explain disease onset; however, many are now considered outdated. For example, H.W. Scheuermann’s original hypothesis that the characteristic alterations in the vertebrae can be attributed to osteonecrosis of the ring apophysis [11] was not corroborated by cadaveric studies by Bick and Copel, who suggested that it is not part of the epiphysis and therefore would have not been involved in the longitudinal growth of the vertebral body [12]. Schmorl’s assumption that the eponymous nodes are the main contributors of the hallmark vertebral alterations of SK has been strongly contested in the literature [13,14,15,16]. Other authors suggested a combination of congenital predisposition of the spine and mechanical factors in adolescence [17,18]. Lambrinudi highlighted the role of tight hamstrings in exerting excessive flexion and stresses on the anterior part of the growing vertebral bodies during bending, thereby contributing significantly to the development of kyphosis [19].

As there is a lack of work in the international literature that examines all the past and current research data of the underlying pathophysiological mechanisms and their correlation with the macroscopic structural deformities during the development of SK, this comprehensive review aims to summarise current knowledge on the fundamental characteristics of SKand the prevailing theories regarding its pathophysiology. Emphasis is placed on the interplay between mechanical factors, spinopelvic parameters, and genetic and molecular contributions, which have seen significant advances in recent years.

## 2. Pathophysiology of Structural Deformities

### 2.1. Mechanical Factors

The increased prevalence of SK among manual labourers who began working at a young age and among high-level athletes [11,20,21], along with the findings of a 1992 study by Revel et al. demonstrating that repetitive strain on vertebral endplates in young rats leads to typical Scheuermann lesions [22], supports the hypothesis that the degree of mechanical stress influences the severity of spinal impairment in SK. Correspondingly, Wassmann in 1951 emphasised the role of increased mechanical stress on the developing spine—particularly from manual labour—as a major aetiological factor in SK. He observed a higher prevalence among Danish army recruits from rural districts with a history of agricultural or heavy labour, referring to SK as an ‘occupational disease’ [23]. In a 1977 paper, Alexander proposed that SK occurs as stress spondylo dystrophy, as a sequelae of traumatic growth stunt and endplate fractures (compression failure) occurring in the vulnerable adolescent spine during rapid growth in puberty [13]. Characteristic structural changes in the affected spinal segments occur subsequently due to abnormal stresses applied on the spine by a prolonged sitting position [13], leading to anterior vertebral failure [24]. Ogden also supports this possible mechanical aetiology, adding that the presence of other concomitant spine pathologies, such us spondylolysis or spondylolisthesis, as well as a partial reversal of vertebral height after conservative treatment further supports SK as a result of abnormal stresses [24]. It is also well known that bracing works in principle by alleviating the forces from the anterior column of the spine [25]. Remodelling of wedged vertebrae after treatment with braces, with long-term maintenance of the result has been documented in the literature [26,27]. Furthermore, especially for skeletally immature patients, wedged vertebrae remodelling has been observed after posterior correction surgery; the authors used Ponte osteotomies and fusion to shorten the posterior column and decrease loads on the anterior part of the vertebrae [28]. These provide strong indications of a significant mechanical component in the pathogenesis of SK. Biomechanical data suggest reduced mineral density in the anterior portion of the thoracic vertebrae in adults [29]. Furthermore, in the standing position, the greatest flexion moments are observed at the apex of the thoracic kyphotic curve [30]. Additionally, the greatest compressive loads during activity occur in the thoracolumbar spine, due to local muscle forces and the presence of thoracic kyphosis [30]. These findings of increased stresses align with the anatomical locations and vertebral regions typically affected in SK. However, since the samples comprise older adult patients, they should be interpreted with caution regarding the adolescent spine.

Similarly, a prematurely fused short sternum might result in excessive compressive forces on the vertebral endplates anteriorly, thereby allowing uneven growth of the VBs with wedging, as described by Fotiadis’ et al. investigative study [31]. The authors examined over 10,000 patients and the 175 Scheuermann patients diagnosed were found to have a statistically significant shorter sternum than healthy controls [31]. However, it remains unclear whether this difference in sternum length is a contributing factor or a consequence of the pathological process underlying SK.

Up to a third of SK patients have been reported to exhibit a coexistent scoliotic deformity; this may occur in different degrees but is usually clinically mild [32,33]. Hurtado-Avilez et al. proposed that vertebral wedging in the coronal plane may underlie the coexistence of scoliosis in these patients, differentiating it from Idiopathic Scoliosis [34]; however, this hypothesis remains unproven. To date, cadaveric studies have primarily focused on anteroposterior vertebral measurements [16,35,36]. Furthermore, the presence of concurrent scoliosis in SK has received limited attention in the literature, particularly regarding its implications for pathophysiology, diagnosis, management, and outcomes. Further research is needed in this direction.

More recent work examining spinopelvic parameters in skeletally immature patients with SK shows these patients exhibit decreased Pelvic Incidence (PI) and Sacral Slope (SS) compared to healthy controls, and those changes remain unchanged into adulthood [37]. Peleg et al. in their cadaveric study found that the sacrum was significantly more horizontally inclined (as measured by sacral anatomical orientation, i.e.,a decrease in SS [38]) in spines that met SK criteria compared to non-affected spines [35]. The authors argued that this horizontal orientation would produce compensatory changes in Lumbar Lordosis (LL) and eventually kyphotic deformity in the thoracic spine with erect posture to maintain sagittal balance; this would increase the pressures on the anterior part of the thoracic vertebrae and lead to the already described hallmark changes [35]. It is unknown if the aforementioned changes in inclination are of a significant magnitude to produce the kyphotic deformity in SK; nor is the developmental stage at which they occurred in the presented cadaveric samples known. A positive association of SS and LL is also observed in patients without spinal pathology in the literature [39]. Furthermore, a positive association between Thoracic Kyphosis (TK) and SS was found by Bezalel et al. in their cross-sectional study of 150 patients [2]. The increased LL found in patients with SK has been thought to be a secondary adaptation in order to maintain sagittal balance, because of the increased TK [2]. Jansen et al. reported a correlation of TK and LL, and between LL and SS, preoperatively in SK patients; after correction of TK there was an associated decrease in LL [40]. Sacral Slope remained unchanged postoperatively, suggesting a static pelvis [40]. A lower PI and Pelvic Tilt has been associated with SK compared to healthy controls in other studies, as well as an association between TK and Cervical and Lumbar Lordosis; based on the latter it was presumed these are compensatory changes produced by TK [40,41,42]. These mechanisms are also distinct in the typical and the atypical form of SK, owing to the different location of the deformity [41]. Other studies have not linked TK and PI or provided evidence for a difference in PI between SK patients and healthy subjects, disputing the role of the pelvis in the pathogenesis of SK [43,44]. Hosman et al. noted that patients with SK and tight hamstrings, which are variably present, may be less able to compensate for changes in sagittal balance as a result of a fixed pelvis, which is thought to occur via the LL or the change in SS [45]. On the other hand, changes in Pelvic Incidence and Sacral Slope have been documented in long term follow-up after posterior fusion surgery for Idiopathic Scoliosis in adolescent patients, presumably as a compensatory mechanism in the sagittal plane since the unfused lower spinal segments remained unchanged [46]. Furthermore, a 2007 study by Gum et al. suggested that for AIS, a change in pelvic orientation in the transverse plane may develop as a compensatory response to the coronal plane spinal deformity. This was attributed to the alignment direction coinciding with the thoracic curve, its relatively smaller magnitude, and the absence of sagittal plane involvement [47].

Other studies regarding spinopelvic alignment in children suggest a more positive sagittal vertical axis in younger children moving backwards until adulthood, as well as a correlation with the LL [48,49,50]. Thoracic kyphosis is thought to normally increase slowly until the 20 years of age, with a corresponding increase in Lumbar Lordosis, and a correlation between LL and SS [51] according to earlier works in the developing spine [52]. A progressively more horizontal Sacrum may be part of the normal spine growth [48,52]. Additionally, the sacrum did not seem to correlate with the SVA [48]. These findings suggest that pelvic changes may occur normally in the developing spine and therefore further research is needed in the determination of their contribution in spinal pathology regarding sagittal alignment.

Evidence of SK in quadrupedal primates hints at a genetic component in the pathogenesis of the disease, indicating that upright posture and mechanical stresses in the thoracic spine by bipedal walking are not solely responsible for the development of kyphosis, as a purely mechanical theory would imply [53].

The interaction between pelvic orientation and spinal curvature is a matter of ongoing research; further investigation is needed to elucidate their role in the pathogenesis of the disease. Compensatory pelvic adaptations have been documented in other spinal deformities to maintain balance and posture; thus, it is plausible that the pelvis may play a similar compensatory role in the sagittal plane deformity seen in SK. Further research is needed to quantify changes in spinopelvic parameters in SK and to determine their association with the characteristic vertebral changes, as current data remain inconclusive. Data derived from studies regarding spinopelvic parameters in SK is presented in Table 1.

### 2.2. Vertebral Body Wedging and Anterior Longitudinal Ligament

**Figure 1:**
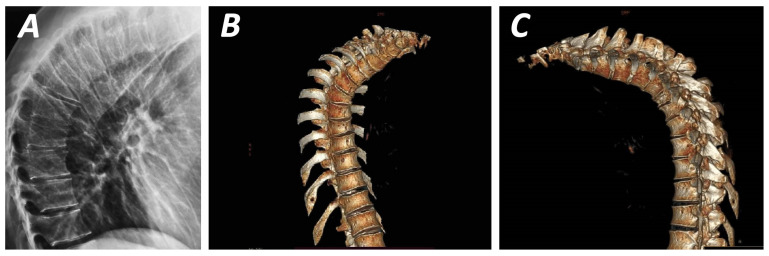
Plainlateral radiograph (**A**) and three-dimensional CT-scan (**B**,**C**) demonstrating the anterior wedged-shaped vertebral bodies in thoracic hyperkyphosis. This figure is from corresponding author’s clinical archives and illustrates the pathological vertebral wedging of thoracic spine.

**Figure 2:**
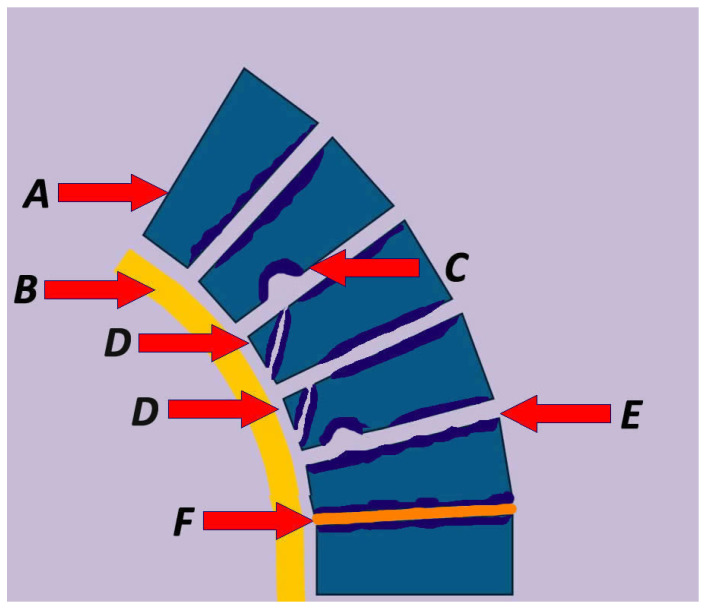
Summary of the pathophysiological characteristic lesions in Scheuermann’s Kyphosis including (A) vertebral wedging, (B) thickening of the anterior longitudinal ligament, (C) Schmorl’s node, (D) impaired ring apophysis, (E) vertebral endplate irregularities, and (F) intervertebral disc abnormalities.

Central to the typical presentation of SK is thoracic spine kyphosis secondary to wedged vertebrae (Figure 1 and Figure 2). Wedging is thought take place during primary ossification and increase during secondary ossification, increasing the forces applied to the endplate, leading to the characteristic difference between anterior and posterior vertebral height [54]. In a skeletal study of 1384 spines, of which 103 met Sorensen’s criteria for the diagnosis of SK, the latter exhibited Schmorl nodes in at least one level, an anterior extension of thoracic vertebrae in 94% of specimens, with an increase in the posterior thoracic vertebral body height compared to controls [16]. A recent study of 150 patients found that 4% of SK patients with an average thoracic kyphosis of 55 degrees exhibited only one anterior wedged vertebra [2]. Another characteristic sign is bone formation opposite a Schmorl node (Edgren-Vaino sign) [6]. Affected vertebrae also exhibit anterior cancellous bone extension, leading to the characteristic anteroposteriorly elongated shape as a result of disrupted endochondral ossification [36]. Mallet et al. described the projection of the ring apophysis under the vertebral endplate in early stages of the disease [55]. Moreover, the thickened or tightened (bowstringing) anterior longitudinal ligament (ALL) limits anterior growth of the vertebra during childhood, contributing to the wedge shape [56,57]. This observation originates from intraoperative findings reported by Bradford and Moe [57]. The biomechanical properties, structure, function, and load patterns of the ALL have been studied in the literature [58,59,60,61,62,63,64,65,66,67,68], revealing high tensile strength, dense collagen fibre composition, low elastin content, and strong bony attachments [62,69]. Furthermore, the high strength of the ALL and elasticity may decrease with disc degeneration, osteoporosis, and ageing [58,63,69] (Figure 2). Disc degeneration may be variably present in SK and a relatively stiffer ligament may exacerbate the deformity (Figure 2).

In a study of midterm human foetuses, Jin et al. demonstrated tight ALL connections to the anterior part of the lumbar vertebral cortex, with close involvement in osteogenesis [70]. It is not known however how the ALL interacts with vertebrae during adolescent growth when SK develops and whether such changes precede vertebral wedging. The significance of the ALL in spinal deformity was also demonstrated in a cadaveric study by Birnbaum et al., which achieved a 4-degree correction per level by transecting the ALL, achieving significant anterior release [71]. Despite these findings, the exact role of the ALL in the pathogenesis of SK has not been thoroughly investigated in the literature.

### 2.3. Schmorl’sNodes and Intervertebral Discs

Schmorl’s Nodes (SN) correspond to a nucleus pulposus herniation in the VB through a weak point of the endplate (Figure 2). They may present anteriorly, posteriorly, as limbus vertebrae—corresponding to anterior disc material herniation and interposition of the annulus between the ring epiphysis and the VB—or as posterior disc bulges, with a wide prevalence ranging from 5 to 70% [8,54,72,73,74,75,76]. Many are discovered as incidental findings [77]. Schmorl’s nodes are present in SK [78], as they develop during secondary ossification. The herniation occurs because of a disruption of the endplate or a weakening of the subchondral bone [76]. Although Peng et al. proposed osteonecrosis as a potential mechanism of herniation based on histologic findings, their sample did not include SK patients [79]. In a study of 100 cadavers (mean age of death 68.2 years) by Pfirrmann and Resnick, 81% of SNs occurred between T7 and L2; the absence of advanced spondylosis features likely suggests that SNs are not a major contributor to spinal degeneration [76].

SNs in SK are typically smaller, involve multiple vertebrae and are located in the posterior part of a lower endplate of the VB. Differentiation between SNs of degenerative, traumatic, or developmental origin is important, though not always straightforward [80]. These lesions have a multifactorial aetiology and are not exclusive to SK. They can occur as a result of axial loading and endplate fracture [77,78,79,80,81]. A lower Bone Mineral Density may be a predisposing factor, and osteoporosis has been investigated in this context, although a definite correlation has not been established [82,83]. Schmorl Node presence is usually associated with disc degeneration [84]. In adult patients, degenerative changes occurring at the apex of the deformity is thought to contribute to pain [32], which may be exacerbated by accompanying spondylolesis or spondylolisthesis [32]. Furthermore, disc degeneration and bulging is observed in MRI scans in almost all patients with typical and atypical SK, affecting multiple levels [33]. Other SK signs are multiple small indentations, irregular endplates, decreased disc height, and decreased signal in T2 images in MRI [8,74]. SNs may appear in isolation, without other Scheuermann lesions, and genetic predisposition has been proposed as an underlying factor in such cases [85]. Reactive sclerotic bone formation is typically present around the node [80]. Finally, other neural abnormalities may be present in patients with SK. In a study of 117 surgically treated patients, 86 of which underwent a preoperative MRI, 2 (2.3%) patients had a low lying conus, 15 (17.4%) had syringomyelia, 49 (56%) had epidural lipomatosis, 31 (36%) had spinal stenosis, and 7 (8.1%) had spondylolysis [86]. These abnormalities may warrant a change in the operative plan [86].

**Figure 3:**
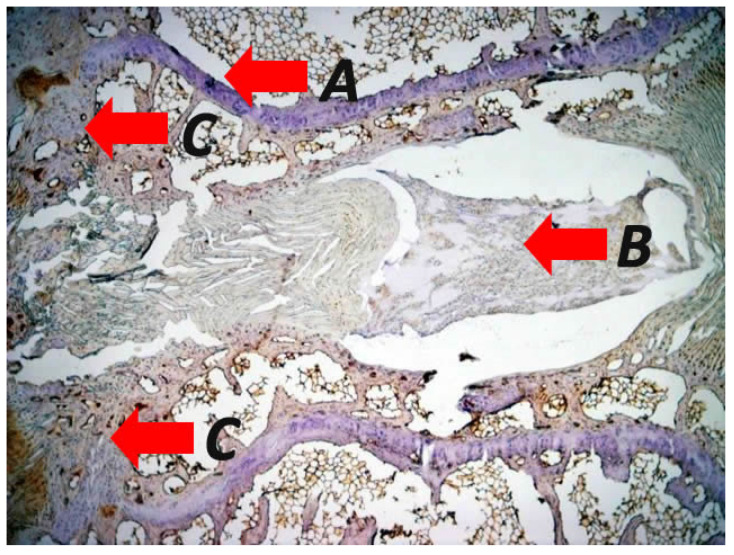
Representative photomicrographs of immunohistological staining in specimens from a mechanically induced vertebral wedging after applying asymmetrical loading at the adjacent vertebras of rats displaying (A) impairment of the intervertebral disc accompanied by tears and delamination of the annulus fibrosus and disruption of the herniated annulus pulposus, (B) irregularities such us structural disruption and altered morphological features of the endplates with (C) increased rate of endochondral ossification during the vertebra wedging process (original magnification 2×). The figure is from the corresponding author’s experimental results and illustrates the pathological concepts of asymmetrical loading at the sagittal plane of the spine.

The literature supports that intervertebral disc (IVD) impairment is observed more frequently in SK than in normal spines (Figure 2 and Figure 3), even in young patients [8,87]. The IVD relies on diffusion for nutrient delivery, with the vertebral cartilage endplate serving as the primary interface for nutrient supply. In SK, the endplate is weakened, leading to defective disc nutrition due to disrupted exchange between the disc and the vertebral endplate [54]. SK is characterised by defective growth of the vertebral cartilage endplate, which is the weakest portion of the disc–vertebra complex and therefore is prone to mechanical failure [88] (Figure 3). Biomechanical data suggest that in patients with Thoracolumbar SK, kyphosis-driven altered biomechanical loads concentrate significantly on the annulus fibrosus part of the IVD compared to healthy controls, thereby predisposing it to degeneration [89] (Figure 3).

In a retrospective study comparing symptomatic patients with thoracolumbar (TL) disc herniation and radiographic evidence of SK to asymptomatic hospital workers with similar radiographic findings, Guo et al. found a greater TL kyphotic angle, irregular endplates, Schmorl nodes and higher BMI in the symptomatic group [87]. Another study, by Liu et al., examined the presence of SK features in symptomatic patients with TL disc herniation; an increased frequency was found compared to patients with low lumbar disc herniation, suggesting an association between the two entities [90]. Kyphosis was mild in the TL group, and the study involved older patients. The herniations caused neurologic deficits; these are considered rare in SK but could have been the result of long-term sequelae of spinal degeneration in the setting of a genetic predisposition influencing SK [90]. A similar association was described by Gille et al. [91]. A case of atypical SK with TL endplate irregularities and an anterior defect of the S1 vertebrae in a gymnast has been reported in the literature [92] (Figure 2).

## 3. Bone Metabolism-Related Factors

Regarding the role of osteoporosis, reports in the literature are conflicting. Bradford and Moe in their histologic study described findings in two patients with SK and hypothesised that the changes could be a result of vertebral osteoporosis [57]. In a prospective study of 10 SK patients, Lopez et al. found that for kyphosis over 45 degrees, the femoral neck and lumbar spine BMD was statistically significantly lower in SK patients than in control subjects, suggesting osteoporosis as a significant comorbidity and/or pathogenetic contributor in the disease [93]. This was measured with dual photon absorptiometry, which is less accurate than DEXA [94] and now considered outdated.

Although childhood/juvenile osteoporosis has been proposed as a factor in the aetiopathogenesis of SK, no causeandeffect correlation has been demonstrated in the literature. Gilsanz et al. demonstrated no evidence of osteoporosis in adolescent patients with SK, emphasising that this aetiological hypothesis remains debatable and warrants further investigation [95]. The authors used quantitative Computed Tomography (CT) to assess the bone density of twenty otherwise healthy adolescents with SK and compared it to twenty controls without underlying health problems, matched for age, sex, and race. Further investigating a possible osteoporosis link, Ashton et al. used Dual-Energy X-ray Absorptiometry (DEXA), to determine the BMD of the lumbar spine in 12 patients (average age of 14.1 years) with SK and then compared the results to control values with data from a children’s hospital [96]. The authors concluded that SK patients exhibited higher BMD than age- and sex-matched controls, though the number of patients was small. Conversely, Popko et al. found lower BMD in the lumbar spine and total body BMD in 9/24 SK patients aged 9–18 years old using DEXA, when compared to control values [97]. Quantitative CT exhibits higher sensitivity in diagnosing osteoporosis; however, its purported advantages over DEXA in accounting for degenerative changes and vertebral calcifications are less relevant in the adolescent population under investigation [98]. Furthermore, the sample sizes of the above studies are relatively small, highlighting the necessity for further research to draw definitive conclusions. Finally, another paper by Gilsanz et al. studying BMD by quantitative CT in over 101 children that underwent trauma related abdominal CT of various ages, failed to prove a relative reduction in BMD in adolescents [99]. This study provided evidence that osteoporosis may not be commonly encountered in the developing spine. Thus, it has direct implications for the pathophysiology of SK, indicating that any osteoporosis contribution may be investigated in the presence of other genetically mediated pathologies, as is analysed later. Data from studies concerning BMD measurement and SK is summarised in Table 2.

In a more recent prospective study of men aged 50–85 years, patients with SK had statistically significant higher whole-body BMD and lower hip BMD, without a statistically significant difference in BMD in other sites [100]. Lastly, in a multicentre European study, Armbrecht et al. found no significant impact of SK on lumbar spine or femoral neck BMD in patients over the age of 50 [6]. However, since these last two studies comprise an older group of patients, data regarding BMD should be interpreted with caution.

Several other factors and their role in the development of SK have been researched. In a small prospective study of 62 tall girls aged 9–18 years old, tall stature was linked to scoliosis and SK with increased frequency compared to the general population [101]. An association between increased height and spinal deformities was also observed in a later study of more than 800,000 adolescents; an increase in deformity severity was also associated with a low BMI [102]. As mentioned earlier, weight and height in SK patients tends to be greater than age- and sex-matched healthy controls, although the relation to the aetiopathogenesis of the disease is unclear [103]. Additionally, growth hormone hypersecretion, defective formation of collagen fibrils with subsequent weakening of the vertebral endplates, dural cysts, trauma, biomechanical stressors such as vitamin A deficiency, poliomyelitis, and epiphysitis have been proposed to fully or partially explain the pathogenetic mechanism of SK [56,104,105,106,107,108]. Furthermore, hormonal changes associated with premature pubarche may aggravate preexisting spinal deformity as in Idiopathic Scoliosis [109]. Finally, although its role is yet unknown, Vitamin D deficiency in SK patients has been linked to worse clinical outcomes [110].

Interestingly, the above clinical findings can be partially elucidated by Frost’s mechanostat theory. This theory examines bone adaptation in response to mechanical loading, whereby bone remodelling is regulated according to the degree of mechanical stimulus, akin to a thermostat—thus termed the “mechanostat” by Frost [111,112]. According to this theory, muscular loads applied to bone trigger adaptive remodelling to maintain ‘carrying capacity’ at a pre-set strain threshold, ultimately influencing osseous morphology [113,114]. Conversely, disuse and lack of muscle force exerted on bone trigger the resorption process, leading to bone loss predominantly adjacent to the marrow [111]. The above feedback mechanism is achieved by the transduction of altered cellular mechanical forces to chemical signals and is influenced by a variety of factors, such as diseases as well as hormonal and nutritional alterations that may take place at various timescales [112,115,116]. It is important to note that Frost advocated for the use of his mechanostat theory to all load-bearing bones, including the human vertebrae. An inherently elevated activation threshold could weaken bones and increase their susceptibility to mechanical stress and fractures [111]. Furthermore, in the adolescent growth phase, bone remodelling and adaptation is associated with weight and muscle growth, i.e., increasing lever forces. However, data regarding the impact on vertebral morphology in the absence of sudden mechanical loading, for example after a fall or after osteoporotic vertebral fractures, are limited. Frost described bone failure in the metaphyseal part of long bones when deforming forces were exerted [111].

Recent literature has highlighted the correlation between muscle–bone interactions and bone development during puberty [117]. Rauch et al. reported that increased Lean Body Mass, a surrogate for muscle force, is associated with increased Bone Mineral Content (BMC) in adolescents. This finding supports the concept that muscle development promotes bone formation, although the potential contribution of genetic factors cannot be excluded [118]. However, many of the primary structural characteristics of SK may be secondary adaptations to abnormal increased loads—for example, anterior cortical extension or bone formation adjacent to Schmorl’s nodes. Nevertheless, it has not been clarified yet if the proposed remodelling changes affect a specific part of the bone or its entirety. As increased thoracic kyphosis during puberty is associated with increased muscular forces, it is reasonable to assume that these mechanical factors play a central role in vertebral reshaping. Other disease alterations have been shown to be a result of aberrant spinal loading, leading to degenerative disease. The roles of muscle imbalance, the variable influence of BMD, and the function of the muscle–bone functional unit in the development of spinal deformity warrant further investigation.

## 4. Genetic Foundation

Various studies have investigated the genetic link and the possible inheritance pattern of SK. Axenovich et al. studied 90 pedigrees of families in Siberia with at least one SK patient and used a transmission probability model to determine the method of inheritance [119]. They concluded that an autosomal dominant inheritance pattern with a major mutant gene could best explain the transmission in the tested families; they also suggested complete penetrance in men and incomplete penetrance in women affected [119]. Prevalence of idiopathic scoliosis was also higher in the families [119]. A major gene contribution was also concluded in a further investigation of 90 SK patients and 385 relatives by Zaidman et al. [120]. In studies on 35,000 twins in the Danish Twin Registry, Damborg et al. reported an incidence of 2.8% for SK and found a high degree of heritability. A higher incidence of SK was observed amongst monozygotic twins, leading the authors to emphasise a significant genetic influence in the manifestation of the disease [121,122]. However, the proportion of overall SK cases attributable to this specific inheritance pattern remains unknown.

Numerous publications highlight the heritability of SK. Indicatively, Kewalramani reported three cases of coexistence of SK and Charcot-Marie-Tooth syndrome, with neurologic deficits, in three generations of a family [123]. In 1978, Halal et al. reported on five families with seemingly autosomal dominant inheritance of SK [124]. Although the various family members had variable expression of the disease and some were asymptomatic, penetrance was high [124]. A similar pattern was described in seven families in a study by McKenzie et al. [125] and in three generations of one family by Findlay et al. [126]. Graat et al. reported SK in monozygotic twins with similar levels of activity, wedged vertebrae and different degrees of thoracic kyphosis (Cobb angle of 74 and 48) [127]. Similar reports of twins with radiographic abnormalities suggestive of SK can be found in the literature, either symptomatic or not [128,129,130]. Van Linthoudt and Revel described a case of twins with identical changes suggestive of SK in the lumbar spine, namely anterior extension of L2, a Schmorl’s Node, (and the Edgren-Vaino sign [6]), of which only one twin was symptomatic, a difference attributed to her participation in rigorous sports activities [17]. Another report associated the inheritance of SK with craniosynostosis over five generations of a family with a dominant pattern [131]. Finally, Dai et al. reported on a family with lumbar SK and Idiopathic Scoliosis with a pattern fitting the described inheritance model [132].

Earlier studies have proposed a predisposing genetic background influencing the quality of matrix components (Type II and Type IX collagen) and chondrocytes [121,122]. SK and hip dysplasia were reported in a young patient with type II Collagenopathy (mutation in COL2A1) treated with periacetabular osteotomy [133]. Thoracic kyphosis with irregular endplates, vertebral wedging and Schmorl nodes, along with early-onset severe osteoarthritis and short metatarsals have been associated with an Arg-75-Cys mutation in the procollagen type II gene (COL2A1) in a family, and a specific mutation in COL9A3 (Trp3 allele) was found in patients reporting low back pain with radiographic Scheuermann’s Kyphosis, disc degeneration and endplate lesions [134,135,136]. Specific alleles in the COL9A2 gene have been linked to Lumbar Disc disease by other studies [136,137]. Duffy and COL1A2 gene association was previously excluded [125]. Another COL2A1 mutation in mice also led to disorganised endochondral ossification and growth plate disturbances, arthritic changes, and caused a type of Congenital Spondyloepiphyseal Dysplasia with a dominant inheritance pattern [138]. Furthermore, a study by Oei et al. showed a single nucleotide polymorphism associated at genome-wide significance level with SK mapping to TLL1 [139]. Although unusual, vertebral alterations similar to lumbar SK were reported in a 32-year-old patient with Osteogenesis Imperfecta and a heterozygous mutation in COL1A2 [140]. The role of the SOX9 gene and the chondroblast receptor have also been proposed as targets of investigation [120].

In a 2001 study by Iba et al., the role of tetranectin in developing musculoskeletal tissues was evaluated by the generation of knockout mice [141]. Tetranectin-deficient mice exhibited rigid kyphotic deformity; a significant proportion (23/60) of the kyphotic mice exhibited structural vertebral changes, namely wedging with reduced vertebral height anteriorly, endplate irregularities and shortened and/or broadened vertebral bodies, without, however, changes in disc spaces or long bones and skeletal muscle [141]. Histologically, these mice showed compression of the anterior intervertebral discs and disorganised, irregular growth plates, which ultimately led to disc protrusions [141]. These changes are similar to structural changes observed in SK patients. Furthermore, BMD measurements excluded a possible osteoporotic aetiology in all mice [141]. Molecular pathways and the role of tetranectin in human disease is a subject of investigation [142]. In contrast, deletion of the Mecom allele in mice led to growth retardation, lumbar osteopenia, lumbar lordosis, scoliosis, leading to thoracic kyphosis—changes that were significantly different from the wild-type counterparts [143]. Furthermore, other changes included disc degeneration, vertebral articulation fusion, and narrow intervertebral spaces as well as structural tendon changes in the mutant mice [143]. In humans, SK patients tend to be taller and weigh more than healthy controls [103]. Furthermore, the severity and timeline of the degenerative changes in the lumbar spine, as well as the involvement of the tendons are unusual for human patients with SK. A different pattern of kyphosis aetiology might be more befitting, and further research is needed to establish this.

## 5. Endochondral Ossification

**Figure 4:**
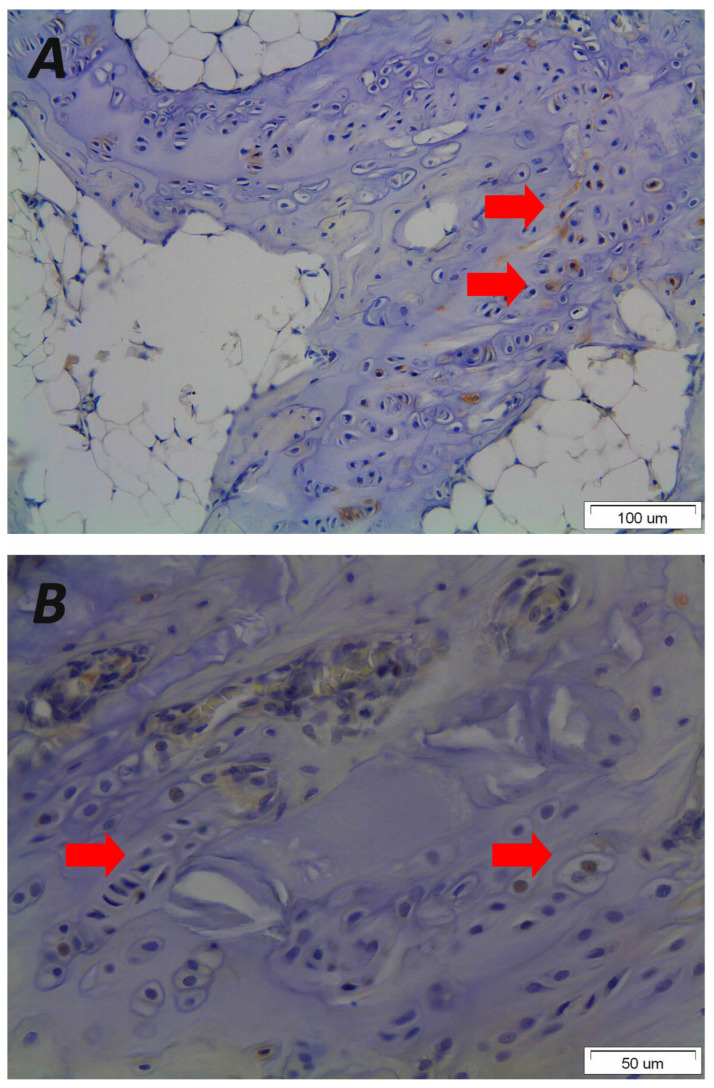
Representative photomicrographs of immunohistological staining from vertebral end plate sections received after asymmetrical loading at the spine of a rat animal experimental model demonstrating that mechanical loading results in disorientated endplates (original magnification 20×) (**A**) that express the osteoblastic growth factor Pleiotrophin in the nucleus and cytoplasm of hypertrophic chondrocytes (red arrows) leading to primary ossification, wedged vertebrae and kyphotic malformation (original magnification 40×) (**B**). Please note the morphological changes and altered orientation of the hypertrophic chondrocytes. The figure is from the corresponding author’s experimental data and illustrates the pathological results of the vertebral endplates after asymmetric loading at the sagittal plane of the spine.

Disorganised endochondral ossification results from defective growth of the cartilage endplate and the induced expression of osteoblastic factors such as Pleiotrophin [136] (Figure 3 and Figure 4). Meanwhile, the relative decrease in collagen has also been hypothesised to lead to an alteration in endplate ossification. Histological studies show disorganised endochondral ossification, reduced collagen level, and increased mucopolysaccharide levels in endplates in the presence of SK, without the presence of necrotic bone [16,144,145]. Aufdermaur and Spycher described areas with absent or diminished collagen fibres, areas of discontinuity/herniation (Schmorl nodes) and adjacent areas with absent or irregular ossification centres in the vertebral endplates of patients showing changes consistent with SK [146]. They concluded that a dysfunction of collagen and ground matrix biosynthesis leading to a growth impairment and weakening of the endplates have a pivotal role in the pathogenesis of SK [146].

These results are consistent with other histologic studies demonstrating disrupted endochondral ossification, similar to changes observed in Blount’s disease [16,106]. The authors regard the vascular and fibroblast proliferation in the affected endplates as a chronic reparative response to chronic traumatic stimuli [16]. Therefore, excessive mechanical stress on a low-quality/weakened vertebral endplate during spinal growth causes disc impairment and disproportional asymmetrical VB growth [120], with resultant classic wedge-shaped VBs that will lead to kyphosis [120]. However, it remains unclear whether these changes precede abnormal mechanical loading of the spine or arise as a consequence thereof. Absent anterior ossification centres and dysplastic epiphyseal cartilage, albeit relatively avascular, with ventral compression of the intervertebral discs, is found in pigs with juvenile kyphosis [147].

In a 2013 study, Zaidman et al. studied morphohistochemical changes in spinal structural elements in patients with SK by isolating chondroblasts from the VB growth plates and subjecting them to morphological, biochemical, and ultrastructural analyses, and by assessing the qualitative and quantitative composition of the growth plates using a culture medium. These changes included disturbance of the structural and chondral organisation of cells and matrix in the VB growth plate, a narrow growth plate, a decrease in chondroitin sulphate content and an increase in keratan sulphate content, and a lower response to oxidation-reduction enzymes in the cytoplasm of chondroblasts. Furthermore, isolated Golgi apparatus enlarged cisterns in the endoplasmic reticulum, with fragmented matrix were also observed in affected vertebral parts [120]. Collagen genes were also well expressed [120].

## 6. Molecular Signalling

Recent studies investigating the underlying mechanisms of the SK focused on the mechanotransduction molecular interactions between Cerebrospinal Fluid (CSF) flow and spinal morphogenesis in vivo [148]. Reissner fibres are contained in the CSF and consist of acellular threads extending from the roof of the third ventricle to the caudal end of the central canal. They are composed of an aggregation of cilia-dependent glycoprotein CSO-spondin [149,150]. Moreover, it was demonstrated that through the vertebrates the CSF-contacting neurons (CSF-cNs) modulate locomotion by projecting the essential glutamatergic components of the locomotor central pattern generators (CPGs) [151]. Additionally, in vitro studies displayed that CSF-cNs express Polycystic Kidney Disease 2-like 1 (PDK2L1), which is a member of the Transient Receptor Potential (TRP) channel superfamily involved in Ca^+2^-signalling in primary cilia, is also a pH and osmolarity sensitive transient receptor channel that forms an intraspinal mechanosensory organ responding to active and passive bending of the spinal cord [152,153]. The critical role of PDK2L1 in the mechanosensory activity of CSF-cNs as well as in the preservation of spinal alignment was examined in *pdk2l1* zebrafish mutants, showing that defective mechanosensory functions of CSF-cNs were associated with severe spinal curvature at the sagittal plane, at the ages between 12 and 19 months of age [150]. The above observations were confirmed by a study by Hardy et al. where the *pdk2l1* zebrafish mutants did not develop spinal deformities at the coronal plane and/or vertebral malformations [154].

Additionally, the spinal morphology in zebrafish is characterised by a kyphotic longitudinal curvature at the thoracic level resembling the human spine [155]. Therefore, to determine the similarities between *pdk2l1* zebrafish mutants and SK, micro-computer tomography analysis was performed based on criteria such as amplitude, the anatomical location of hyper-kyphotic curves, curve pattern, and sagittal balance, which define this pathology in human subjects [154]. Indeed, homozygous mutant experimental zebrafish displayed a remarkable increase in thoracic kyphotic Cobb’s angles similar to ratios reported in patients suffering from SK [154]. Moreover, the curve pattern index, calculated by the ratio between the kyphotic height and the spino-spinal length, revealed significant deformation at the sagittal plane in homozygous *pdk2l1* mutants compared to wild-type mutants [154]. The evaluation of the Harrington index, used to assess curve progression, was also elevated in the *pdk2l1* mutants and was accompanied by higher lordotic curves at the caudal spine compared to their wild-type siblings [154]. Interestingly, male *pdk2l1* mutants exhibited a 1.5-fold higher median kyphotic angle compared to female experimental animals, mirroring the sex-related distribution observed in humans [154]. Therefore, the asymmetric mechanosensory activation of CSF-cNs due to defective Reissner fibre signalling, which leads to selective activation of the sensory neurons on the concave side, may be caused either by the direct contact of Reissner fibres and CSF-cNs or increased CSF flow at the apical extension of the cells [153]. These findings are in agreement with the clinical findings of increased frequency of epidural lipomatosis that produce epidural and CSF pressure, indicating a possible correlation between CSF circulation and the development of SK [156,157].

## 7. Limitations of the Study

To the best of our knowledge, this review represents the most up to date synthesis of genetical, molecular, histological, and macroscopic data of possible pathophysiological mechanisms of SK. Since its first description in the 1920s, numerous conflicting theories have been proposed for the pathogenesis of SK. This work has several strengths and limitations. It presents a comprehensive review of the literature, incorporating both historical and contemporary data relevant to the pathogenesis of SK. This includes studies from the past century, as well as novel theories stemming from advances in molecular and genetic pathways. Moreover, the studies’ designs and methods were heterogeneous as different protocols were used and no standardised methods were applied. Furthermore, the limited experimental clinical evidence, and the small patient’s sample, raises concerns about the definitive involvement of the described pathophysiological mechanisms. Finally, a language bias could be present as only studies written in English were reviewed.

## 8. Conclusions

It is well established that a strong genetic component leads to the characteristic changes in the spines of SK patients. However, a unified pathophysiological model remains lacking. When biomechanical data on stress distribution and vertebral bone mineral density are considered, it is notable that the characteristic deformities of SK—particularly those occurring during the rapid growth phase of adolescence—are localised to regions of the thoracic spine that experience the highest mechanical stress and have inherently weaker structural integrity. This anatomical overlap supports the hypothesis that a genetic predisposition may render specific spinal regions more susceptible to mechanical loading, resulting in the hallmark deformities of the disease.

Conflicting data regarding osteoporosis, outdated techniques, and small sample sizes continue to hinder definitive conclusions. Furthermore, while certain gene mutations are known to produce Scheuermann-like deformities, SK cannot be solely attributed to these genetic variants, as the full spectrum of associated mutations is not consistently observed in SK patients. Further research should be undertaken in this direction.

## Figures and Tables

**Table 1:** Studies investigating the association between Scheuermann’s Kyphosis and Spinopelvic parameters.

Study	Number of Patients (N)	Age (Mean)	Male/Female Ratio	Diagnosis	Mean Kyphosis	Intervention	Follow Up (Months)	Outcome
Peleg et al., 2016 [35]	SK Group: 183 Control group: 185 normal skeletons	N/A	N/A	Sorensen’s criteria	N/A	Cadaveric study/Radiographic parameter analysis	N/A	SAO: SK group: 44.44 ± 9.7°, Control group: 50 ± 9.9° (*p* < 0.01). Sacrum more horizontally inclined in SK patients.
Tyrakowski et al., 2015 [37]	Total N: 66 Group 1: skeletally mature SK: 33 Group 2: skeletally immature SK: 33	Skeletally mature 22.7 (16.1–47.4) Skeletally immature: 14.1 (11–16.3)	Skeletally mature: 2 Skeletally immature: 3.12	Sorensen’s criteria	Skeletally mature: 56° (3–81) Skeletally immature: 57.8° (13–96)	Radiographic parameter analysis/No intervention	N/A	Skeletally mature pts: PI: 39.4 ± 8.9 PT: 7.3 ± 9.4 SS: 32.1 ± 9.2 Skeletally immature pts: PI: 36.7 ± 8.1 PT: 3.8 ± 7.5 SS: 32.8 ± 9.2 No significant difference between PI, PT, SS, LL SK patients have lower PI and SS than non- patients.
Jansen et al., 2006 [40]	30	28	1.3	Sach’s Criteria (TK > 45° and at least one wedged vertebra ≥ 5°)	80°	PSF: -Harrington rods: 5 Pts -Cotrel-Dubousset 25 Pts Anterior release: 29 Pts	12	Maximum kyphosis (±SD): preop: 80° (±7) postop: 47° (±9) Maximum lordosis (±SD): preop: 72° (±12) postop: 59° (±11) Mean L5-S1, SS unchanged pre- and postoperatively Avg correction of kyphosis: 2.3x Avg correction of lordosis Postoperative correlation of kyphosis versus lordosis (R = 0.591, *p* = 0.001)
Jiang et al., 2014 [41]	Total N: 115 SK Pts: 55 (subdivided in Thoracic Kyphosis and Thoracolumbar kyphosis subgroups) Healthy controls: N:60	SK Group: 14.2 (10–18) Control group: 14.2 (11–18)	SK Group: 3.23 Control group: N/A	Sorensen’s criteria	45.6° ± 24.3	Radiographic parameter analysis/No intervention	N/A	SK group: PI: 32° ± 10.8 Control group: 45° ± 10.8 (*p*: 0.001) SK group: PT: 0.2° ± 11.0 Control group: 11° ± 9.2 (*p*: 0.001) SK Thoracic Kyphosis subgroup: TK strongly correlated with LL (r = −0.792, *p*\0.001) and PT (r = 0.551, *p* = 0.008). PI related to PT (r = 0.514, *p* = 0.014) and SS (r = 0.564, *p* = 0.006). No correlations were found between LL and SS. For thoracolumbar kyphosis group: LL correlated with SS (r = −0.641, *p*\0.001) and PI (r = −0.365, *p* = 0.037).
Loder, 2001 [42]	N:34	15.5	1.12	Sorensen’s criteria	65° ± 12	Radiographic parameter analysis/No intervention (Preoperative Radiographs)	N/A	Cervical lordosis correlated with LL (Cobb angle, r^2^ = 0.17, *p* = 0.024) No correlations between cervical lordosis and TK or sacral inclination.
Cahill et al., 2015 [43]	Total N: 97 SK group: N:47 Control group: N:50 (from database)	SK group: 16.1 Control group: 13.5	SK group: 2.61 Control group: 0.28	N/A	65.5° ± 13.4	Radiographic parameter analysis (Multi-centre study)/Retrospective Preoperative radiographic parameter analysis	24	SK group: PI: 41.8° ± 12.0, PT: 7.3° ± 8.1, SS: 34.5° ± 9.5 Control group: PI: 45.5° ± 8.5, PT: 8.4 ° ± 6.7, SS: 37.1° ± 8.5 Above results not statistically significant different between studied populations. SK group: LL 66.3° ± 12.9 Control group LL: 55.1° ± 11.9 (*p* < 0.001) T5-12 kyphosis and C7-SVA correlated with LL (*p* < 0.05) PI directly correlated with LL in both groups (*p*!0.005) Greatest Cobb kyphosis in SK did not significantly correlate with PI or LL.
Lonner et al., 2007 [44]	N:78	16.7 (9–27)	7.54	Sorensen’s criteria	78.8°	Pts divided as follows: -Combined Anterior and Posterior Surgery: 42 Pts -PSF: 36 Pts (Multi-centre study) Radiographic parameter analysis	34.8 ± 16.8	TK: Whole group preop: 78.8° ± 11.6 (55–106), final follow-up: 51.4° ± 12.3 (27–82) Correlation of LL and PI both before surgery (*p* < 0.001) and at follow-up (*p* = 0.000). LL correlated with TK at final follow-up (*p* < 0.02) No correlation between LL and TK before surgery (*p* = 0.23). TK did not correlate with PI. LL statistically significant increase in both groups, between the first postoperative visit and final follow-up. Whole group LL: Postop: 46.5 ± 12.3, Follow-up 51.7 ± 13.8 (*p* = 0.053)
Hosman et al., 2003 [45]	Total N: 33 Group 1: Pts w/tight hamstrings N:16 Group 2: Pts w/nonlight hamstrings N:17	Group 1: 24.9 Group 2: 26.6	N/A	N/A	Group 1: 78.1° Group 2: 79.3°	Surgical correction and fusion, Radiographic parameter analysis	54 (24–98.4)	Kyphosis correction: Group 1: 27.2°, Group 2: 22.2° (*p*: 0.14) LL Reduction: Group 1: −10.3°, Group 2: −10° (*p*: 0.93) SS shift: Group 1: 0.19°, Group 2:6.3° (*p*: 0.0001) Preoperatively sagittal imbalanced: statistically non-significant between groups Postoperatively sagittal imbalanced: Group 1: 8/16, Group 2: 1/16 (0.036) Group 2: Greater pelvic and lumbar ROM compared to group 1

**Table 2:** Studies that analyse the vertebral metabolic alterations in Scheuermann’s Kyphosis.

Study/Country	Number of Patients (N)	Age Mean and Range (in Years)	Mean Kyphosis (in Degrees)	Method	Site of Measurement	Results
Lopez et al., 1988, United States [93]	SK group n = 10 Control group n = 7 Total n = 17	SK:16 (14–19) Control group:16	64° (35–85°) Control group: no spinal pathology	Dual Photon Absorptiometry/Single Photon Absorptiometry	Right femoral neck, L2–L4/Distal radius	Mean BMD: SK group 0.975, Control group 1.130 (*p*: 0.025) (DPA) Femoral neck BMD: SK group 1.00, Control Group 1.22 (*p* < 0.005) (DPA) Subgroup with kyphosis > 45° (n = 7) BMD Lspine: 0.913 (*p* < 0.005), BMD femoral neck: 0.983 (*p* < 0.005) (DPA) Distal radius BMD: SK group: 0.689, Control group: 0.748. Not reaching statistical significance (SPA).
Gilsanz et al., 1989, United States [95]	SK group n = 20 Control group n = 20 Total n = 40	SK group: 15.6 ± 1.9 Control group: 15.6 ± 2.1	56°(45–79) Control group: no spinal deformity	Quantitative CT	L-Spine	SK Group: 199.2 ± 18.7 mg/cm^3^ of mineral equivalent Control Group 193.7 ± 17.4 mg/cm^3^ of mineral equivalent. Not significantly different.
Ashton et al., 2001, Australia [96]	SK pts = 12	SK group: 14.08 (11–16) Control group: data from 335 pts 14.5	N/A	DEXA	L2–L4	Mean Lumbar Z-score 1.55 (0.0–3.9)
Popko et al., 1997, Poland [97]	SK n= 24	N/A. Control group: population database	N/A	DEXA	L2–L4	In 9/24 SK pts lower Total Skeleton BMD and L2–L4 BMD compared to population data.

## Data Availability

No new data were created or analysed in this study. Figure 1 illustrates the pathological vertebral wedging of thoracic spine. Figure 3 and Figure 4 demonstrates the established concept of pathological results of vertebral and endplate morphological alterations after the application of asymmetrical loading at the sagittal plane, respectively. Data will be made available upon reasonable request.
